# Electrogenerated Chemiluminescence Biosensor for Quantization of Matrix Metalloproteinase-3 in Serum via Target-Induced Cleavage of Oligopeptide

**DOI:** 10.3390/bios14040181

**Published:** 2024-04-08

**Authors:** Manping Qian, Yu Zeng, Meng Li, Qiang Gao, Chengxiao Zhang, Honglan Qi

**Affiliations:** Key Laboratory of Analytical Chemistry for Life Science of Shaanxi Province, School of Chemistry and Chemical Engineering, Shaanxi Normal University, Xi’an 710062, China; manpingqian@snnu.edu.cn (M.Q.); lemon@snnu.edu.cn (M.L.); gaoqiang@snnu.edu.cn (Q.G.);

**Keywords:** electrogenerated chemiluminescence, matrix metalloproteinase 3, gold nanoparticles, biosensor, cyclometalated iridium(III) complex, peptide

## Abstract

A highly sensitive and selective electrogenerated chemiluminescence (ECL) biosensor was developed for the determination of matrix metalloproteinase 3 (MMP-3) in serum via the target-induced cleavage of an oligopeptide. One ECL probe (named as Ir-peptide) was synthesized by covalently linking a new cyclometalated iridium(III) complex ([(3-pba)_2_Ir(bpy-COOH)](PF_6_)) (3-pba = 3-(2-pyridyl) benzaldehyde, bpy-COOH = 4′-methyl-2,2′-bipyridine-4-carboxylic acid) with an oligopeptide (CGVPLSLTMGKGGK). An ECL biosensor was fabricated by firstly casting Nafion and gold nanoparticles (AuNPs) on a glassy carbon electrode and then self-assembling both of the ECL probes, 6-mercapto-1-hexanol and zwitterionic peptide, on the electrode surface, from which the AuNPs could be used to amplify the ECL signal and Ir-peptide could serve as an ECL probe to detect the MMP-3. Thanks to the MMP-3-induced cleavage of the oligopeptide contributing to the decrease in ECL intensity and the amplification of the ECL signal using AuNPs, the ECL biosensor could selectively and sensitively quantify MMP-3 in the concentration range of 10–150 ng·mL^−1^ and with both a limit of quantification (26.7 ng·mL^−1^) and a limit of detection (8.0 ng·mL^−1^) via one-step recognition. In addition, the developed ECL biosensor showed good performance in the quantization of MMP-3 in serum samples, with a recovery of 92.6% ± 2.8%–105.6% ± 5.0%. An increased level of MMP-3 was found in the serum of rheumatoid arthritis patients compared with that of healthy people. This work provides a sensitive and selective biosensing method for the detection of MMP-3 in human serum, which is promising in the identification of patients with rheumatoid arthritis.

## 1. Introduction

Matrix metalloproteinases-3 (MMP-3), a member of the matrix metalloproteinases, can degrade extracellular proteoglycans and type IV collagen; activate the activities of MMP-1, MMP-8 and MMP-9; accelerate articular cartilage aging; and exacerbate osteoarthritis (OA) and rheumatoid arthritis (RA). MMP-3 is mainly present on the synovium and cartilage surfaces of knee joints in patients with OA and RA [1,2]. It has been found that the MMP-3 levels in the serum and joint fluid of patients with OA and RA are significantly higher than those of healthy people [3,4]. Therefore, MMP-3 is regarded as a biomarker for elucidating the molecular mechanisms of clinical diagnoses such as OA and RA. Enzyme-linked immunosorbent assay (ELISA) is commonly used in hospitals for the detection of MMP-3 [5]. Other immunoassays, such as electrochemical immunoassay [6,7] and fluorescent immunoassay [8], are also reported for the detection of MMP-3, though both of these are limited by tedious operations. Biosensing methods using oligopeptides as a molecular recognition substrate have been well-documented to be particularly attractive for various analytical purposes due to advantages of convenience, cost-effective synthesis and resistance to the harsh environments of the oligopeptides [9,10]. It was reported that a specific peptide (GVPLSLTMGKGG) could be used as a molecular recognition substrate for MMP-3 assay in plasma and serum [11]. To date, the fluorescent method [12] and plasmon resonance method [13] were reported for the determination of MMP-3 in plasma and serum. It is still needed to develop new biosensing methods for the detection of MMP-3 in real samples with high selectivity and sensitivity in clinical diagnosis.

Electrogenerated chemiluminescence (electrochemiluminescence, ECL) occurs at or near the electrode surface, resulting from a series of electrochemical and chemiluminescence reactions [14]. Due to their advantages of low background and high sensitivity, ECL biosensing methods have received increasing attention in the field of clinical diagnosis [15]. For peptide-based ECL biosensing, there are usually three biosensing types used in analyte assays, including an oligopeptide binding mode, a target-induced cleavage of oligopeptide mode and an oligopeptide phosphorylation mode. Lots of peptide-based ECL biosensing methods have been reported for different targets using different biosensing modes. For example, in our lab, we recently developed ECL biosensing methods for MMP-9 assay based on a target-induced cleavage of oligopeptide mode [16]. Several other ECL biosensing methods from other research groups have been developed for MMP-2 assay [17], thrombin assay [18,19], tryptase assay, caspase-3 assay [20] and PSA assay [21] via a target-induced cleavage mode, and MMP-14 assay [22] via an oligopeptide binding mode. For example, Zhang et. al. developed an ECL biosensor for the detection of thrombin by employing a thrombin-specific peptide as a molecular recognition substrate in 100-fold diluted serum samples [18]. Among them, the target-induced cleavage mode is promising for the detection of protease. When the target is incubated with an ECL probe (an ECL emitter labeled peptide), the target-induced cleavage of the ECL probe occurs, leading to the ECL intensity change. Satisfactory analytical performance was obtained for the target assay. Additionally, in the target-induced cleavage mode, an ECL probe composed of an ECL emitter and a molecular recognition substrate is a key issue for the development of ECL biosensing methods. Our group have synthesized (pq)_2_Ir(dcbpy) (pq = 2-phenylquinoline, dcbpy = 2,2′-bipyridyl-4,4′-dicarboxylic acid) as an ECL emitter for MMP-9 assay [16]. Fan’s group synthesized nitrogen-doped graphene quantum dots-Ru(bpy)_3_^2+^-doped silica nanoparticles as ECL materials for MMP-2 assay via the target-induced cleavage mode [17]. Zhang’s group utilized Ru(bpy)_3_^2+^-doped-silica nanoparticles as ECL materials for thrombin assay via the target-induced cleavage mode [18]. Luo’s group employed CuInZnS/ZnS quantum dots and Au@Luminol as dual ECL materials for thrombin assay [19] via the target-induced cleavage mode. New ECL emitters with high ECL efficiency and good solubility in aqueous solution are highly required for peptide-based ECL biosensing methods in terms of the sensitivity of the analytical method. From what we know, there are no reports on the detection of MMP-3 based on electrogenerated chemiluminescence biosensing methods using an oligopeptide as a molecular recognition substrate.

**Scheme 1:**
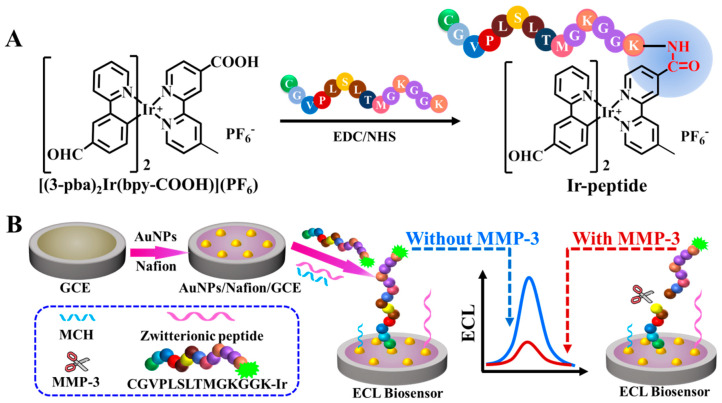
The schematic diagrams for the synthesis of Ir-peptide (**A**) and the fabrication of the peptide-based ECL biosensor for the detection of MMP-3 (**B**).

Here, we present an ECL biosensor for the quantization of MMP-3 by employing a new cyclometalated iridium(III) complex through the target-induced cleavage mode. As shown in Scheme 1A, a new aldehyde group and carboxyl group bearing the cyclometalated iridium(III) complex ([(3-pba)_2_Ir(bpy-COOH)](PF_6_), abbreviated as Ir, 3-pba = 3-(2-pyridyl) benzaldehyde, bpy-COOH = 4′-methyl-2,2′-bipyridine-4-carboxylic acid) was designed and synthesized as an ECL emitter. In addition, [(3-pba)_2_Ir(bpy-COOH)](PF_6_) was covalently linked with the specific peptide (Cys-Gly-Val-Pro-Leu-Ser-Leu-Thr-Met-Gly-Lys-Gly-Gly-Lys, CGVPLSLTMGKGGK) as a molecular recognition substrate to get an ECL probe (Ir-peptide) according to reference [11]. To further improve the sensitivity of the biosensor, gold nanoparticles (AuNPs) were employed not only as signal amplification materials for the ECL system ([(3-pba)_2_Ir(bpy-COOH)](PF_6_)-tri-n-propylamine (TPA)), but also as immobilizing platforms for the oligopeptide. The immobilizing platforms were fabricated by modifying a glassy carbon electrode (GCE) with both Nafion and AuNPs to form a AuNPs/Nafion/GCE. An ECL biosensor was prepared by self-assembling the Ir-peptide, 6-mercapto-1-hexanol (MCH) and zwitterionic peptide (CEKEKEK) onto the AuNPs/Nafion/GCE (Scheme 1B). In the presence of MMP-3, the ECL intensity was decreased via the target-induced cleavage of the oligopeptide. This is the first case of an ECL biosensing method for MMP-3 assay with high sensitivity and selectivity via a cleavage mode and AuNPs signal amplification.

## 2. Materials and Methods

### 2.1. Materials and Apparatus

IrCl_3_·3H_2_O was obtained from Xi’an Shengyi New Material Technology Co., Ltd. (Xi’an, China). 3-(2-pyridyl) benzaldehyde (3-pba), 4′-methyl-2,2′-bipyridine-4-carboxylic acid (bpy-COOH), MMP-3 and TPA (98%) from Sigma-Aldrich (St. Louis, MO, USA); CEKEKEK and CGVPLSLTMGKGGK from Shanghai Apeptide Co., Ltd. (Shanghai, China); and ultrapure water (Millipore Milli-Q, 18.2 MΩ·cm) were used in this work. Other chemicals are listed in the Supplementary Materials. MPI-E ECL detector (Xi’an Remax Analysis Instruments Co., Ltd., Xi’an, China), FluoroMax-4 spectrometer (HORIBA, Piscataway, NJ, USA), UV–Vis spectrophotometer (UV-2450, Shimadzu Corporation, Kyoto, Japan) and CHI 660E electrochemical workstation (Shanghai Chenhua Instruments Co., Ltd., Shanghai, China) were used. A glassy carbon electrode (GCE, 2.0 mm diameter), a platinum counter electrode and an Ag/AgCl reference electrode (saturated KCl) were used in this work.

### 2.2. Synthesis of [(3-pba)_2_Ir(bpy-COOH)](PF_6_) Labeled Peptide

[(3-pba)_2_Ir(bpy-COOH)](PF_6_) was synthesized through two steps [23,24], in which 3-pba was used as the main ligand and bpy-COOH was used as the auxiliary ligand (Supplementary Materials, the synthetic route shown in Scheme S1). First, the dichloro-bridged iridium dimer [(3-pba)_2_Ir(μ-Cl)]_2_ was synthesized by adding IrCl_3_·3H_2_O and 3-pba (C^N ligand) to a mixture of 2-ethoxyethanol and H_2_O. Second, the reaction between [(3-pba)_2_Ir(μ-Cl)]_2_, bpy-COOH and KPF_6_ occurred in 30 mL CH_3_OH/CH_2_Cl_2_ (1/1, *v*/*v*) solution. After purification with dilution and recrystallization, the pure [(3-pba)_2_Ir(bpy-COOH)](PF_6_) was obtained and characterized by ^1^H NMR and mass spectrometry.

^1^H NMR (600 MHz, DMSO) δ 9.86 (d, *J* = 4.8 Hz, 1H), 9.85 (d, *J* = 4.8 Hz, 1H), 9.11 (s, 1H), 9.01 (s, 1H), 8.49–8.40 (m, 4H), 7.99 (ddd, *J* = 34.7, 22.3, 6.8 Hz, 4H), 7.71 (d, *J* = 5.8 Hz, 1H), 7.66 (d, *J* = 5.7 Hz, 1H), 7.62 (d, *J* = 5.6 Hz, 1H), 7.48 (d, *J* = 5.9 Hz, 1H), 7.37–7.33 (m, 2H), 7.24 (t, *J* = 6.6 Hz, 1H), 7.20 (t, *J* = 6.5 Hz, 1H), 6.37 (dd, *J* = 16.2, 7.9 Hz, 2H), 2.50–2.49 (d, *J* = 6.0 Hz, 3H). ESI-MS (*m*/*z*): calculated for [(3-pba)_2_Ir(bpy-COOH)]^+^ [M]^+^ 771.1580; found: 771.1592.

The Ir-peptide was synthesized following the process mentioned in reference [16]. First, [(3-pba)_2_Ir(bpy-COOH)](PF_6_) was activated by stirring a mixture of 1.9 mg 1-(3-dimethylaminopropyl)-3-ethylcarbodiimide, 1.2 mg n-hydroxy succinimide and 100 μL of 1.0 mM [(3-pba)_2_Ir(bpy-COOH)](PF_6_) for 1 h at 18 °C. After that, 800 μL of 10 mM phosphate buffer (PB, pH 7.40), 100 μL of the activated [(3-pba)_2_Ir(bpy-COOH)](PF_6_) and 100 μL of 1.0 mM peptide were mixed together, left overnight and then dialyzed with 10 mM PB (pH 7.40) PB at 4 °C.

### 2.3. Fabrication of ECL Biosensor and Measurement of MMP-3

The AuNPs were synthesized based on reference [25] and the AuNPs/Nafion/GCE was prepared according to our previous work [16]. The modified electrode was then immersed into the mixture solution of 5.0 μM Ir-peptide and 16.7 μM zwitterionic peptide for 5 h, followed by immersion into 1.0 mM MCH for 30 min (Scheme 1B). As a control, a gold electrode-based ECL biosensor (MCH/zwitterionic peptide@Ir-peptide/gold electrode) was also fabricated by immersing the gold electrode into the mixture solution of 5.0 μM Ir-peptide and 16.7 μM zwitterionic peptide for 5 h, then into 1.0 mM MCH for 30 min.

For MMP-3 measurement, the ECL biosensor was immersed into samples containing MMP-3 at 37 °C for 40 min. ECL measurement was done in 0.10 M PBS (pH 7.40) containing 50 mM TPA, using triangular wave potential scan with a scan rate of 0.10 V·s^−1^. PMT is biased at −900 V. MMP-3 was quantified by the change of ECL intensity.

## 3. Results and Discussion

### 3.1. Characterization of Ir-Peptide

Here, an aldehyde and carboxyl group bearing a cyclometalated iridium(III) complex, [(3-pba)_2_Ir(bpy-COOH)](PF_6_), was synthesized as a new ECL emitter. [(3-pba)_2_Ir(bpy-COOH)](PF_6_) was generated from the reaction of [(3-pba)_2_Ir(μ-Cl)]_2_ with a bpy-COOH auxiliary ligand, according to general procedure (see Supplementary Materials). The mass spectrum (MS) (Figure S1) and ^1^H NMR spectrum (Figure S2) of [(3-pba)_2_Ir(bpy-COOH)](PF_6_) were both recorded in order to verify the molecular structure of it. Six peaks centered at *m*/*z* 769.1566, *m*/*z* 770.1603, *m*/*z* 771.1592, *m*/*z* 772.1624, *m*/*z* 773.1671 and *m*/*z* 774.1681, respectively, were observed (Figure S1), which are attributed to [(3-pba)_2_Ir(bpy-COOH)]^+^ (calculated, 769.1554, 770.1588, 771.1580, 772.1611, 773.1645 and 774.1678). The resonance signals of protons at 9.86 ppm and 9.85 ppm, corresponding to the aldehyde protons; 9.11 ppm–6.37 ppm, corresponding to the aromatic protons; and 2.50–2.49 ppm, corresponding to the methyl protons of [(3-pba)_2_Ir(bpy-COOH)](PF_6_) (Figure S2) indicate the successful synthesis of [(3-pba)_2_Ir(bpy-COOH)](PF_6_).

**Figure 1:**
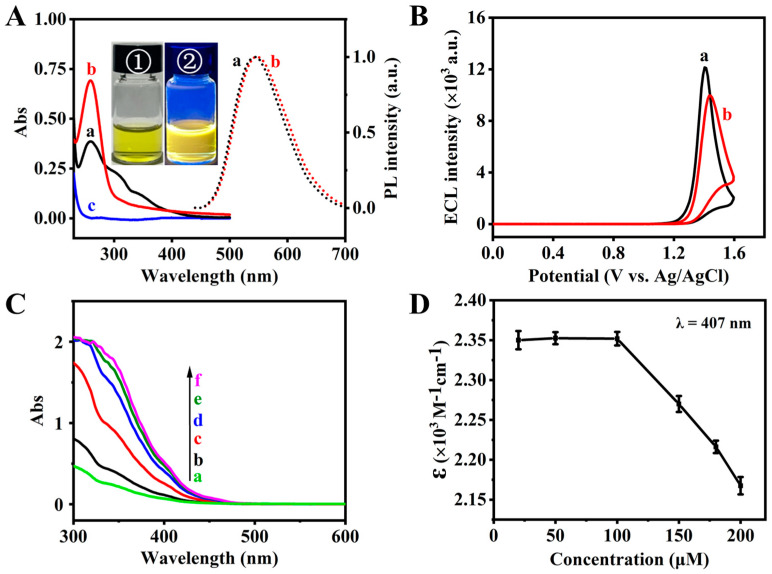
(**A**) UV–Vis absorption spectra (solid line) of 10 μM Ir (a), Ir-peptide (b) and 0.1 mM peptide (c) and photoluminescence spectra (dot line) of 5.0 μM Ir (a) and Ir-peptide (b) in 10 mM PB-DMSO (*v*/*v* = 99/1, pH 7.40), excitation wavelength is 407 nm; inset, pictures of 1.0 mM Ir solution under daylight (①) and UV light (365 nm, ②). (**B**) ECL intensity vs. potential profiles of 0.5 μM Ir (a) and 0.5 μM Ir-peptide (b) in 0.10 M PBS (pH 7.40) containing 50 mM TPA. Scan rate, 0.10 V·s^−1^, PMT, −900 V. (**C**) UV–Vis absorption spectra of [(3-pba)_2_Ir(bpy-COOH)](PF_6_) at different concentrations in 10 mM PB-DMSO (*v*/*v* = 99/1, pH 7.40). Concentrations: a, 20 μM; b, 50 μM; c, 100 μM; d, 150 μM; e, 180 μM; f, 200 μM. (**D**) Molar absorption coefficient at 407 nm of [(3-pba)_2_Ir(bpy-COOH)](PF_6_) at different concentrations. Error bars represent standard deviation of three independent experiments.

Firstly, the solubility, optical and ECL behaviors of [(3-pba)_2_Ir(bpy-COOH)](PF_6_) were studied. Typical absorption peaks at 259, 312, 348 and 407 nm (Figure 1A, a, solid line) and an obvious fluorescence emission with maximum wavelengths at 545 nm (Figure 1A, a, dot line) were obtained for [(3-pba)_2_Ir(bpy-COOH)](PF_6_) in aqueous solution. The UV and the visible regions of the absorption peaks should be due to intra-ligand (π-π*) and MLCT transitions. The low-energy MLCT band at 407 nm and the fluorescence emission at 545 nm of [(3-pba)_2_Ir(bpy-COOH)](PF_6_) are blue-shifted compared to that of [(bt)_2_Ir(bpy-Fc)]PF_6_ (bt = 2-phenylbenzothiazole, bpy-Fc = 4-ferrocenecarbonyl hydrazinocarbonyl-4′-methyl-2,2′-bipyridine, 413 nm) [26], indicating the extent of the π-acceptor strength of the CN-ligand with the aldehyde group compared to the CN-ligand with the thiazole group. The optical behaviors, yellowish-green color and yellow emission can also be clearly visualized under daylight (①, inset, Figure 1A) and UV light irradiation (②, inset, Figure 1A).

For ECL signal reagents used in biosensing applications, good solubility in aqueous solutions is required. The solubility of [(3-pba)_2_Ir(bpy-COOH)](PF_6_) was checked using optical methods. Absorption peaks at 259, 312, 348 and 407 nm gradually increased when increasing the concentration of [(3-pba)_2_Ir(bpy-COOH)](PF_6_). When the concentration was up to 150 μM, the absorption value remained nearly stable (Figure 1C). The relationship between the molar absorption coefficient at 407 nm and the [(3-pba)_2_Ir(bpy-COOH)](PF_6_) concentration in 10 mM PB-DMSO (*v*/*v* = 99/1, pH 7.40) showed that the molar absorption coefficient at 407 nm remained in the concentration range of 20 μM–100 μM [(3-pba)_2_Ir(bpy-COOH)](PF_6_). When the concentration of [(3-pba)_2_Ir(bpy-COOH)](PF_6_) was higher than 100 μM, the molar absorption coefficient at 407 nm decreased (Figure 1D). So, the solubility is thought to be 100 μM. Compared with the reported solubility of [(bt)_2_Ir(bpy-Fc)]PF_6_ (6.0 μM) [26] and Ir(ppy)_3_ (ppy = 2-phenylpyridine, 0.1 μM) [27] in references, this [(3-pba)_2_Ir(bpy-COOH)](PF_6_) shows good solubility because of the presence of the –COOH group.

The ECL behavior of [(3-pba)_2_Ir(bpy-COOH)](PF_6_)-TPA was further studied. As shown in Figure 1B, ECL emission of [(3-pba)_2_Ir(bpy-COOH)](PF_6_)-TPA with a peak potential at +1.40 V can be clearly observed (Figure 1B, a). The ECL efficiency of the [(3-pba)_2_Ir(bpy-COOH)](PF_6_)-TPA system is calculated to be 252.3% compared with that of the typical Ru(bpy)_3_^2+^-TPA system. Then, [(3-pba)_2_Ir(bpy-COOH)](PF_6_) can be used as a promising ECL emitter.

The successful synthesis of Ir-peptide was verified by the UV–Vis, fluorescence and ECL techniques. A characteristic peak at 259 nm was observed for Ir-peptide (Figure 1A, b (solid line)), while there was no absorption in the 250 nm and 500 nm range for peptide (Figure 1A, c). From the fluorescence emission spectra, it can be seen that Ir-peptide shows an obvious fluorescence emission at 550 nm (Figure 1A, b (dot line)), similar to that of Ir alone. From the ECL behavior, an ECL emission at +1.45 V was obtained for Ir-peptide-TPA (Figure 1B, b), while an ECL emission at +1.40 V was obtained for Ir-TPA (Figure 1B, a). Compared with that of Ir alone, a little redshift of PL emission from 545 nm to 550 nm and a positive shift of ECL potential from 1.40 V to 1.45 V were obtained for Ir-peptide. These results indicated that [(3-pba)_2_Ir(bpy-COOH)](PF_6_) was successfully labeled onto the oligopeptide.

### 3.2. Construction of the ECL Biosensor

The ECL biosensor was constructed by self-assembling Ir-peptide onto the AuNPs/Nafion/GCE. Cyclic voltammograms (CVs) and electrochemical impedance spectroscopy (EIS) were used to characterize the construction process of the ECL biosensor. The recorded peak currents of the modified electrode at each step show a trend of first decreasing from 69.69 μA at the bare GCE to 14.93 μA at the AuNPs/Nafion/GCE, then increasing to 25.31 μA at the Ir-peptide/AuNPs/Nafion/GCE and finally decreasing to 11.65 μA at the MCH/zwitterionic peptide@Ir-peptide/AuNPs/Nafion/GCE (Figure S3A). Meanwhile, the corresponding EISs at different steps show that the charge transfer resistance (Ret) increases from 37.93 Ω at bare GCE to 10,330 Ω at AuNPs/Nafion/GCE, then decreases to 3106 Ω after Ir-peptide modification, and finally increases to 6532 Ω after blocking with zwitterionic peptide and MCH (Figure S3B, Table S1), indicating the successful construction of the ECL biosensor.

**Figure 2:**
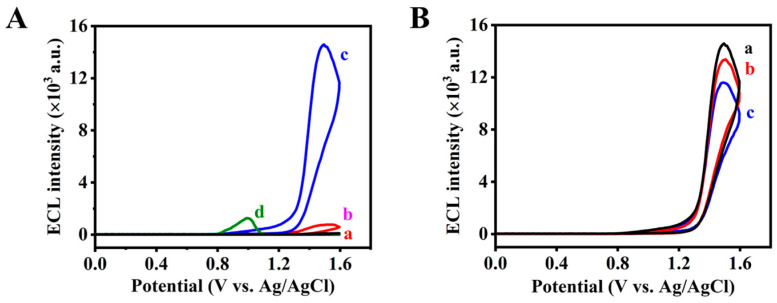
(**A**) ECL intensity vs. potential profiles obtained at different electrodes. (a) Ir-peptide/GCE; (b) Ir-peptide/Nafion/GCE; (c) Ir-peptide/AuNPs/Nafion/GCE; (d) Ir-peptide/gold electrode, respectively. (**B**) ECL intensity vs. potential profiles of the ECL biosensor before (a) and after incubation with 10 ng·mL^−1^ MMP-3 (b) and 30 ng·mL^−1^ MMP-3 (c), respectively. ECL measurement conditions, 0.10 M PBS (pH 7.40) containing 50 mM TPA. Scan rate, 0.10 V·s^−1^, PMT, −900 V.

In this work, AuNPs are not only used as an immobilizing platform for oligopeptides, but are also employed to amplify the ECL signal for the Ir-TPA system. Typical transmission electron microscopy of AuNPs shows the average diameter of ~13 nm (Figure S4). Firstly, the electrochemical surface areas between gold electrode, GCE, Nafion/GCE, AuNPs/GCE and AuNPs/Nafion/GCE were checked using K_3_[Fe(CN)_6_] as an electroactive probe [28]. Different electrochemical surface areas were obtained from AuNPs/GCE (*A* = 0.0220 cm^2^), bare gold electrode (*A* = 0.0174 cm^2^), bare GCE (*A* = 0.0214 cm^2^), Nafion/GCE (*A* = 0.0100 cm^2^) and AuNPs/Nafion/GCE (*A* = 0.0112 cm^2^), demonstrating that AuNPs can effectively increase the electrochemical surface area. Additionally, the ECL signals at different electrodes (Ir-peptide/GCE, Ir-peptide/Nafion/GCE, Ir-peptide/AuNPs/Nafion/GCE, Ir-peptide/gold electrode) were recorded to confirm the amplification function of AuNPs and the stability function of Nafion. In the presence of TPA, the ECL intensity of the ECL biosensor is about 14,588 a.u., while the ECL intensity at the Ir-peptide/Nafion/GCE is only about 729 a.u. (Figure 2A). The ECL intensity of the Ir-peptide/GCE is about 104 a.u., while the ECL intensity of the Ir-peptide/gold electrode is about 1213 a.u. By comparing the ECL intensities of different electrodes, higher ECL intensity was obtained from Ir-peptide/AuNPs/Nafion/GCE (Figure 2A). Additionally, the reproducibility of Ir-peptide/AuNPs/Nafion/GCE was further tested. The good reproducibility of the ECL biosensor was obtained with 2.3% of relative standard derivation (RSD) from one ECL biosensor for ten continuous cycles (Figure S5A) and 5.3% of RSD from six different ECL biosensors for one cycle (Figure S5B). Only one time can be used as a control for the Ir-peptide/gold electrode, although relative ECL intensity can be obtained (Figure S6). Therefore, more Ir-peptide can be modified on the GCE by an Au-S bond between a Cys residue of Ir-peptide and AuNPs. Moreover, Nafion, as the anchoring agent, was employed to make AuNPs firmly modify onto the GCE. Then, AuNPs/Nafion/GCE was employed as the base electrode for the fabrication of the ECL biosensor.

**Figure 3:**
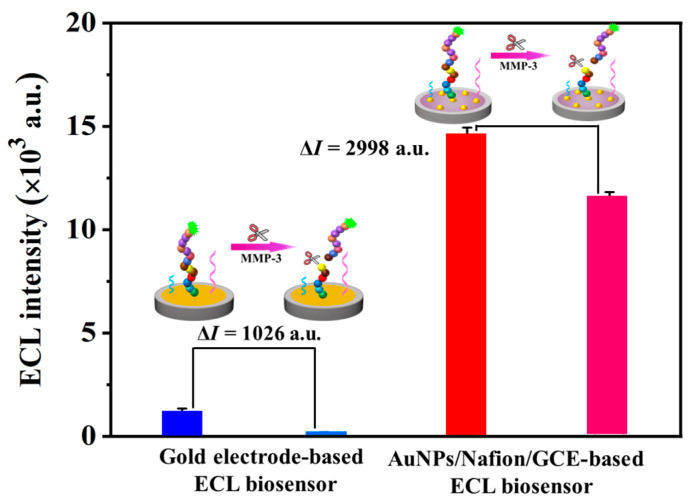
ECL intensity of the two types of ECL biosensors (marked in blue and light blue: the gold electrode-based ECL biosensor; marked in red and pink: the AuNPs/Nafion/GCE-based ECL biosensor) in the absence (blue and red) and presence (light blue and pink) of 30 ng·mL^−1^ of MMP-3. Inset, the schematic diagram of the gold electrode-based ECL biosensor and the AuNPs/Nafion/GCE-based ECL biosensor for the detection of MMP-3. Error bars represent the standard deviation of three independent experiments.

It is reported that MMP-3 can cleave the peptide bond between S and L in a specific peptide of GVPLSLTMGKGG [11]. Then, an ECL probe (Ir-peptide, CGVPLSLTMGKGGK-Ir) is synthesized. The probe on the electrode surface can emit light when there is TPA in solutions. When reacting with MMP-3, the ECL probe can be cleaved and part of the ECL probe can leave from the electrode surface; then the ECL intensity is decreased. The feasibility of the constructed ECL biosensor for MMP-3 assay is then studied. In the absence of MMP-3, there is a strong ECL emission at +1.50 V (14,588 a.u.) from the ECL biosensor. When the concentration of MMP-3 increases from 10 ng·mL^−1^ to 30 ng·mL^−1^, the ECL intensity decreases from 13,374 a.u. to 11,590 a.u. (Figure 2B). Additionally, in order to illustrate the function of gold nanoparticles on the electrode surface, a gold electrode-based ECL biosensor for MMP-3 assay was also studied. As shown in Figure 3 and Figure S7, the ECL intensity at the gold electrode-based ECL biosensor decreased from 1213 a.u. (blank) to 703 a.u. for MMP-3 (10 ng·mL^−1^) and further to 187 a.u. for MMP-3 (30 ng·mL^−1^). The comparison of the ECL responses to MMP-3 at two types of ECL biosensors is listed in Figure 3. As seen from Figure 3, there is a slight decrease in ECL intensity (Δ*I* = *I*_0_ − *I*_s_ = 1026 a.u. for 30 ng·mL^−1^ MMP-3) at the gold electrode-based biosensor (*I*_0_ = 1213 a.u.), while there is a great decrease in ECL intensity (Δ*I* = 2998 a.u. for 30 ng·mL^−1^ MMP-3) and a higher ECL intensity at the AuNPs/Nafion/GCE-based biosensor (*I*_0_ = 14,588 a.u.). Then, the AuNPs’ amplification capability is obvious. The constructed biosensor is promising for the quantization of MMP-3 based on target-induced cleavage of the oligopeptide and using the signal amplification of AuNPs.

### 3.3. Analytical Performance for MMP-3

**Figure 4:**
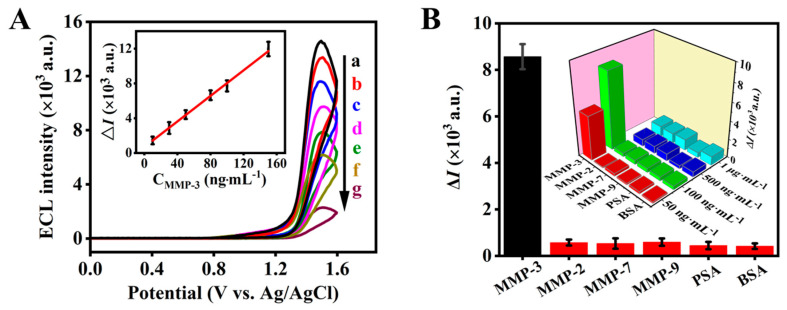
(**A**) ECL intensity vs. potential profiles of the ECL biosensor in response to different concentrations of MMP-3 (a, 0; b, 10 ng·mL^−1^; c, 30 ng·mL^−1^; d, 50 ng·mL^−1^; e, 80 ng·mL^−1^; f, 100 ng·mL^−1^; g, 150 ng·mL^−1^). Inset, calibration curve. (**B**) ECL responses of the ECL biosensor to different model interferents including 100 ng·mL^−1^ MMP-3, MMP-2, MMP-7, MMP-9, PSA and BSA. Inset, ECL responses of the ECL peptide-based biosensor to MMP-3 and other interferents at different concentrations. Condition for the ECL measurement is in 0.10 M PBS (pH 7.40) containing 50 mM TPA. Scan rate, 0.10 V·s^−1^, PMT, −900 V. Error bars represent the standard deviation of three independent experiments.

Since the self-assembly process of the ECL probe and the cleavage reaction between the oligopeptide and MMP-3 have significant impacts on the analytical performance of the biosensor, the times for these two processes are further optimized. For the ECL probe assembly, high ECL intensity is obtained up to 5 h. The cleavage reaction between the modified ECL probe (Ir-peptide) and 150 ng·mL^−1^ MMP-3 finishes after 40 min. In this work, the optimal times for the ECL probe assembly and MMP-3 cleavage are 5 h and 40 min, respectively (Figure S8). Under the optimal conditions, the ECL biosensor displays quantitative responses to different concentrations of MMP-3. Within the MMP-3 concentration ranging from 10 ng·mL^−1^ to 150 ng·mL^−1^, the ECL responses decrease with the increase in MMP-3 concentration (Figure 4A). The linear regression equation is Δ*I* = 73.5 *C* (ng·mL^−1^) + 691.9 (R^2^ = 0.9912). The limit of quantification (LOQ = 10*s*_bl_/*S*, in which *s*_bl_ is standard deviation of the blank signal and *S* is the slope of the calibration curve of MMP-3) and limit of detection (LOD = 3*s*_bl_/*S*) are estimated to be 26.7 ng·mL^−1^ and 8.0 ng·mL^−1^, respectively. It is reported that the MMP-3 levels in the healthy people are located within the range of 29.0 ± 12.7–64.5 ± 29.4 ng·mL^−1^ and at 224.6 ± 237.3–246.4 ± 267.7 ng·mL^−1^ in patients with RA [29]. The obtained limit of quantification (26.7 ng·mL^−1^) is lower than the cutoff value of MMP-3 (70.5 ng·mL^−1^) for diagnosis of severe RA [30]. Good sensitivity is beneficial to MMP-3 assays of complex real samples, since the sample volume can be small and have high dilution.

For real sample analysis, the selectivity is also important. The ECL biosensor’s responses to MMP-2, PSA, BSA, MMP-7 and MMP-9 as interferents at different concentrations were also investigated. For 50 ng·mL^−1^ MMP-3 and interferents, there is an obvious ECL response for MMP-3 (Δ*I* = 4856 a.u.), but there are no ECL responses to other interferents (Δ*I* < 300 a.u.). When the concentration increased to 100 ng·mL^−1^, the ECL response to these interferents was negligible (Δ*I* < 600 a.u., Figure 4B), while a highly obvious response change (Δ*I* = 8395 a.u.) was obtained for 100 ng·mL^−1^ MMP-3. When the concentration of these tested interferents increased to 1.0 μg·mL^−1^, a small ECL change was observed for these tested interferents (Δ*I* < 1200 a.u., Figure 4B, inset). Therefore, the constructed ECL biosensor has good selectivity for the assay of MMP-3, which is promising for the diagnosis of RA.

### 3.4. Sample Analysis

The feasibility of the developed ECL biosensor in practical applications was finally investigated. It is reported that the concentration of MMP-3 in the serum of healthy people is within the range of 29.0 ± 12.7–64.5 ± 29.4 ng·mL^−1^, while a higher concentration of MMP-3 (224.6 ± 237.3–246.4 ± 267.7 ng·mL^−1^) is found in the serum of patients with RA (Table S2) [29]. We tested the MMP-3 in serum samples from three healthy people and three early RA patients (Patient 1, female, age 57; Patient 2, male, age 62; Patient 3, female, age 55), provided by the Hospital of Shaanxi Normal University. Original serum samples have strong effects on the ECL signal of the ECL biosensor (Figure S9). Considering the linear range for the detection of MMP-3 in this work, the recovery of MMP-3 was done by spiking 50 ng·mL^−1^ of MMP-3 in the 20-fold diluted healthy people serum. It was effective, with 92.6% ± 2.8%–105.6% ± 5.0% of recoveries in the 20-fold diluted healthy people serum (Table S3). Then, the ECL biosensor was employed for the detection of MMP-3 in different serum samples of patients with RA. Three patient serum samples are analyzed in this work; 11.2 ± 1.7 ng·mL^−1^, 16.4 ± 1.6 ng·mL^−1^ and 14.5 ± 2.1 ng·mL^−1^ for MMP-3 are calculated for 20-fold diluted serums from the three patients in this work based on the calibration curves in Figure 4A (Table 1). The concentrations of MMP-3 in the original patient serum samples were 224.0 ± 34 ng·mL^−1^, 328 ± 32 ng·mL^−1^ and 290.0 ± 42 ng·mL^−1^. To further enhance credibility, the ELISA method—taken as the standard method—was performed. The obtained results indicate that there is no significant difference between the developed ECL biosensing method and the ELISA method using Student’s *t*-test. We did not detect the concentration in healthy people because of the high detection limit of the developed ECL method. By using ELISA, 4.1 ± 0.2, 3.7 ± 0.3 and 3.8 ± 0.4 ng·mL^−1^ were calculated for the 20-fold diluted serums from the three healthy people (Table S3). Although there is no difference between the MMP-3 expression levels in the unprocessed serum of healthy individuals and patients in reference [11], in our work, the increase in MMP-3 in patients with RA is obvious, consistent with that reported in reference [29,30]. Therefore, the developed method can be applied to determine MMP-3 levels in clinical tests.

The storage stability of the ECL biosensor was further studied. The good storage stability was confirmed by the negligible change (less than 7.4%) in the ECL intensities of the ECL biosensor during 7-day storage in a refrigerator at 4 °C (Figure S5C). In addition, the ECL biosensor can be fabricated within 5.5 h (including 30 min modified time of AuNPs/Nafion onto the GCE, as well as 5 h self-assembly time of Ir-peptide on the ECL biosensor) and MMP-3 can be detected within 1 h (including 40 min cleavage time, less than 5 min detection time) by the use of the fabricated ECL biosensor. Compared with classic ELISA and other reported biosensing methods (Table S4), the measurement of MMP-3 using this method is relatively time-saving. The application of the constructed ECL biosensor for real sample assay—during which the new Ir(III) complex ([(3-pba)_2_Ir(bpy-COOH)](PF_6_)) as an ECL emitter, gold nanoparticles for signal amplification and one-step target-induced cleavage of an oligopeptide are employed—is feasible.

## 4. Conclusions

Here, we reported the first case of a simple, sensitive and selective peptide-based ECL biosensor for the determination of MMP-3 in serum by employing the target-induced cleavage mode. The synthesized [(3-pba)_2_Ir(bpy-COOH)](PF_6_) could be used as a new promising ECL signal reagent with good solubility, high ECL efficiency and an effective linking group (–COOH group). Taking advantage of the MMP-3-induced cleavage of the oligopeptide, the amplification of the ECL signal with AuNPs and the high sensitivity of the ECL method, MMP-3 was determined with a limit of quantification (26.7 ng·mL^−1^) and limit of detection (8.0 ng·mL^−1^). A good recovery (92.6% ± 2.8%–105.6% ± 5.0%) in 20-fold diluted serum samples was obtained via one-step recognition. MMP-3 assay in clinical samples was achieved and the patients with RA were identified. It was found that the level of MMP-3 in RA patients was higher than that in healthy people. This work demonstrates that the ECL peptide-based biosensor is promising in the point-of-care testing of MMP-3 in biological samples.

## Figures and Tables

**Table 1:** Analytical results of MMP-3 in the 20-fold diluted serum of RA patients by using ELISA and the developed ECL biosensor.

Samples	ELISA (ng·mL^−1^)	Content (ng·mL^−1^)
Patient 1 (Early RA, female, age 57)	11.8 ± 0.3	11.2 ± 1.7
Patient 2 (Early RA, male, age 62)	18.4 ± 0.5	16.4 ± 1.6
Patient 3 (Early RA, female, age 55)	15.6 ± 0.3	14.5 ± 2.1

## Data Availability

All original data in this study are included in the article and the Supplementary Materials.

## Supplementary Materials

The following supporting information can be downloaded at: https://www.mdpi.com/article/10.3390/bios14040181/s1, Methods: Synthesis of AuNPs [25] and fabrication of AuNPs/Nafion/GCE [16]; Synthesis of Ir [23,24]; Scheme S1: The synthetic route for Ir; Figure S1: ESI-MS spectra of Ir; Figure S2: ^1^H NMR spectrum of Ir; Figure S3: Characterization of modified electrodes using CVs and Nyquist plots of electrochemical impedance spectra; Figure S4: TEM image of AuNPs; Figure S5: ECL intensity vs. time curve of one ECL biosensor obtained from continuous potential scanning or six independent ECL biosensors for one scan and effect of storage time on the ECL intensity of the ECL biosensors; Figure S6: ECL intensity vs. potential profiles of one gold electrode-based ECL biosensor for continuous potential scanning over ten cycles; Figure S7: ECL intensity vs. potential profiles of gold electrode-based ECL biosensor before and after incubation with MMP-3; Figure S8: The effects of the self-assembly time of Ir-peptide and the cleavage time of MMP-3 on the ECL intensity of the ECL biosensor; Figure S9: ECL intensity vs. potential profiles of the ECL biosensor in the absence and presence of original serum samples; Table S1: Parameter values obtained from the fit of the impedance spectra represented with the equivalent circuit; Table S2: MMP-3 levels in the serum of patients [29]; Table S3: Analytical results of MMP-3 in 20-fold diluted serum of healthy people, measured using ELISA and the developed ECL biosensor; Table S4: Comparison of different reported methods for the determination of MMP-3 [8,11,12,31].
