# Supplementary material for: Electrogenerated Chemiluminescence Biosensor for Quantization of Matrix Metalloproteinase-3 in Serum via Target-Induced Cleavage of Oligopeptide
Source: Biosensors (Basel). 2024 Apr 8;14(4):181. doi: 10.3390/bios14040181 (PMC11047963; doi:10.3390/bios14040181)
Supplement: Supplementary file 1 [file biosensors-14-00181-s001.zip › biosensors-2830635-supplementary.pdf]

Supplementary Information

Electrogenerated Chemiluminescence Biosensor for Quantization of Matrix Metalloproteinase-3 in Serum via Target-Induced Cleavage of Oligopeptide

Manping Qian, Yu Zeng, Meng Li, Qiang Gao, Chengxiao Zhang, Honglan Qi*

Key Laboratory of Analytical Chemistry for Life Science of Shaanxi Province, School of Chemistry and Chemical Engineering, Shaanxi Normal University, Xi'an, 710062, P.R. China

*Corresponding author. Tel.: +86-29-81530726; Fax: +86-29-81530727.

E-mail: honglanqi@snnu.edu.cn.

Materials and methods

Materials and Reagents

Matrix metalloproteinase-2 (MMP-2), matrix metalloproteinase-7 (MMP-7), matrix metalloproteinase-9 (MMP-9), bovine serum albumin (BSA) and prostate-specific antigen (PSA) were purchased from Sigma-Aldrich (St. Louis, MO, USA). In addition, 6-Mercapto-1-hexanol (MCH), Nafion@11 (5.0 wt%), 4-aminophenylmercuric acetate (APMA), N-Hydroxysuccinimide (NHS), 1-(3-dimethylaminopropyl)-3-ethylcarbodiimide hydrochloride (EDC) and dialysis (500 MWCO) were obtained from Tokyo Chemical Industry Co., Ltd. (Tokyo, Japan). Furthermore, 10 mM phosphate buffer solution (PB, 0.0018 M NaH_2PO4 and 0.0082 M Na_2HPO4, pH 7.40) and 0.10 M phosphate buffer solution (PBS, 0.018 M NaH_2PO4, 0.082 M Na_2HPO4 and 0.10 M KCl, pH 7.40) were used inthis work.

Apparatus

Nuclear magnetic resonance spectrometer (Bruker Advance III 600 spectrometry, Karlsruhe, Germany) and electrospray ionization mass spectrometer (ESI-MS, Bruker Maxis UHR-TOF, Karlsruhe, Germany) were used forcharacterization of [(3-pba)$_2$Ir(bpy-COOH)](PF$_6$). JEM-2100 transmission electron microscope (JEOL, Tokyo, Japan) was used for characterization of gold nanoparticles (AuNPs).

Synthesis of AuNPs and fabrication of AuNPs/Nafion/GCE

The synthesis of AuNPs [25] and the fabrication of AuNPs/Nafion/GCE followed our previous process [16]. In brief, gold nanoparticles were prepared by citrate reduction of $HAuCl_4$ in aqueous solution. Next, 100 mL of 0.01% $HAuCl_4$ was brought to reflux and then 4.0 mL of 1% sodium citrate was introduced while stirring. The gold nanoparticle suspension was then kept boiling for another 30 min and left to cool to room temperature. The concentration of AuNPs was estimated to be 1.6×10^{-9} M according to the absorbance at 520 nm ($\varepsilon = 2.7 \times 10^{8}$ mol·L^{-1}·cm^{-1}, 13 nm). Next, 100 μL of 0.5% Nafion solution was mixed with 200 μL AuNPs and sonicated for 30 min. After, 9.0 μL of the mixture of Nafion and AuNPs was drop-coated onto the cleaned glassy carbon electrode (GCE) surface to obtain AuNPs/Nafion/GCE.

Synthesis of [(3-pba)$_2$Ir(bpy-COOH)](PF$_6$)

[(3-pba)$_2$Ir(bpy-COOH)](PF$_6$) was synthesized in two steps. First, the

chloride-bridged dimer $[(3\text{-pba})_2Ir(\mu\text{-}Cl)]_2$ was synthesized using 3-pba as the main ligand according to reported procedures [23,24]. Then, 120 mg of $[(3\text{-pba})_2Ir(\mu\text{-}Cl)]_2$ (0.10 mmol), 47.1 mg of bpy-COOH (0.22 mmol) and 184 mg KPF_6 were dissolved in 30 mL CH_3OH/CH_2Cl_2 (1/1, *v*/*v*) solvent. After degassing, the reaction mixture was stirred for 24 h at 70 ℃ under nitrogen conditions. After cooling to room temperature, the solvent was removed by reduced pressure. Silica column purification with CH_3OH/CH_2Cl_2 (1/1, *v*/*v*) eluent was employed to obtain the pure product.

Supporting Figures and Tables

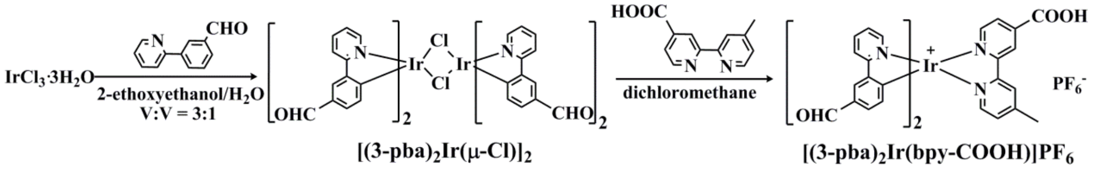

Scheme S1. The synthetic route for [(3-pba)$_2$Ir(bpy-COOH)](PF$_6$).

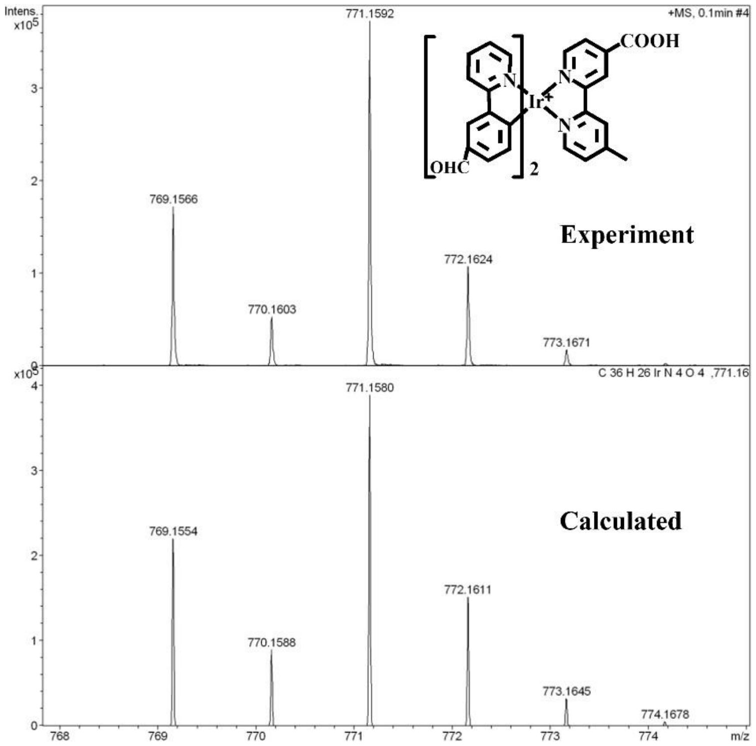

Figure S1 Experimental and calculated ESI-MS spectra of [(3-pba)$_2$Ir(bpy-COOH)]$^+$, [M]$^+$ 771.1592 (calculated, 771.1580).

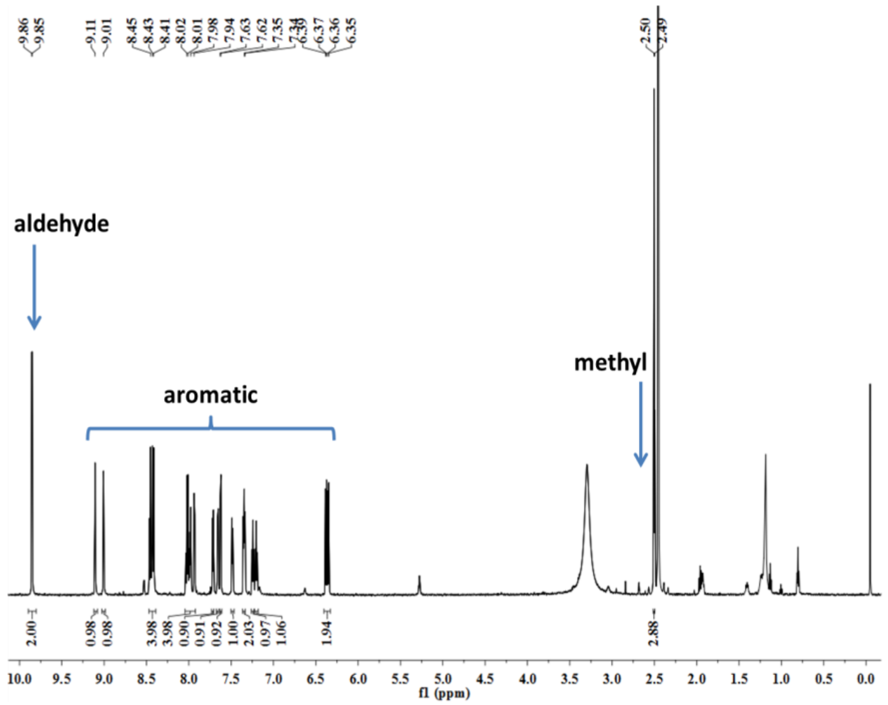

Figure S2 ^{1}H NMR spectrum of [(3-pba)$_2$Ir(bpy-COOH)](PF$_6$) in DMSO-d_6. (600 MHz).

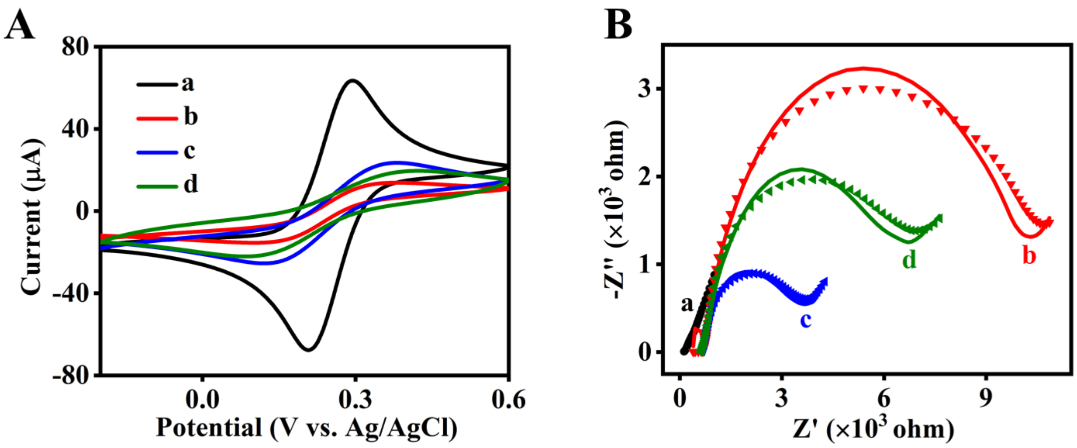

Figure S3 Characterization of modified electrodes using cyclic voltammograms (CVs) (A) and Nyquist plots of electrochemical impedance spectra (B). (a) GCE, (b) AuNPs/Nafion/GCE, (c) Ir-peptide/AuNPs/Nafion/GCE and (d) MCH/zwitterionic peptide@Ir-peptide/AuNPs/Nafion/GCE. The measurement conditions: 0.10 M PBS (pH 7.40) containing 5.0 mM $K_3[Fe(CN)_6]$ and 5.0 mM $K_4[Fe(CN)_6]$. (A) Scan rate, 0.10 $V \cdot s^{-1}$. (B) The biased potential of +0.22 V, the frequency from 100 kHz to 0.1 Hz and the amplitude of 5.0 mV.

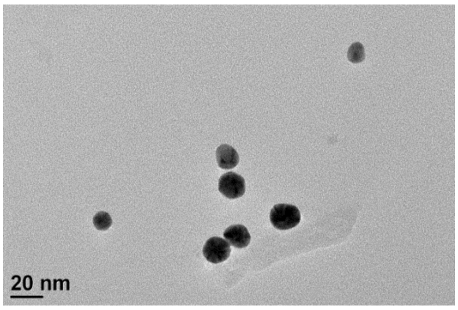

Figure S4 TEM image of AuNPs.

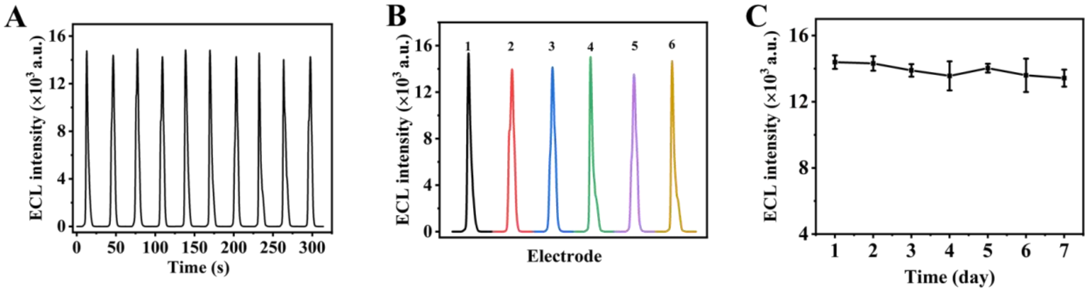

Figure S5 (A) ECL intensity vs. time curve of one ECL biosensor obtained from continuous potential scanning over ten cycles. Scan rate, 0.10 V·s^{-1}. (B) ECL intensity of six independent ECL biosensors. (C) Effect of the storage time on the ECL intensities of the ECL biosensors. Error bars represent the standard deviation of three independent experiments.

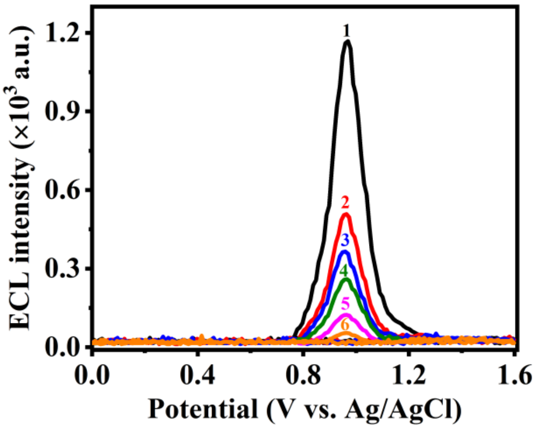

Figure S6 ECL intensity vs. potential profiles of one Ir-peptide/gold electrode for continuous potential scanning over six cycles. ECL measurement conditions, 0.10 M PBS (pH 7.40) containing 50 mM TPA. Scan rate, 0.10 V·s^{-1}, PMT, −900 V.

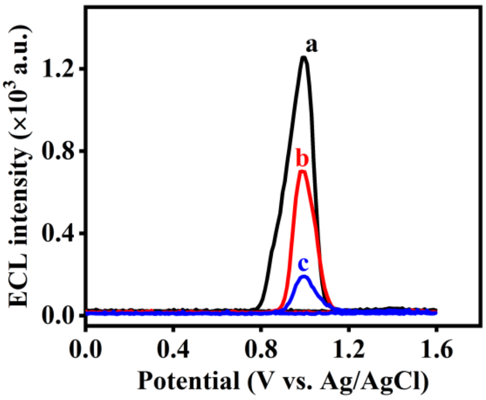

Figure S7 ECL intensity vs. potential profiles of gold electrode-based ECL biosensor before (a) and after incubation with 10 ng·mL^{-1} MMP-3 (b) and 30 ng·mL^{-1} MMP-3 (c), respectively. ECL measurement conditions, 0.10 M PBS (pH 7.40) containing 50 mM TPA. Scan rate, 0.10 V·s^{-1}, PMT, −900 V.

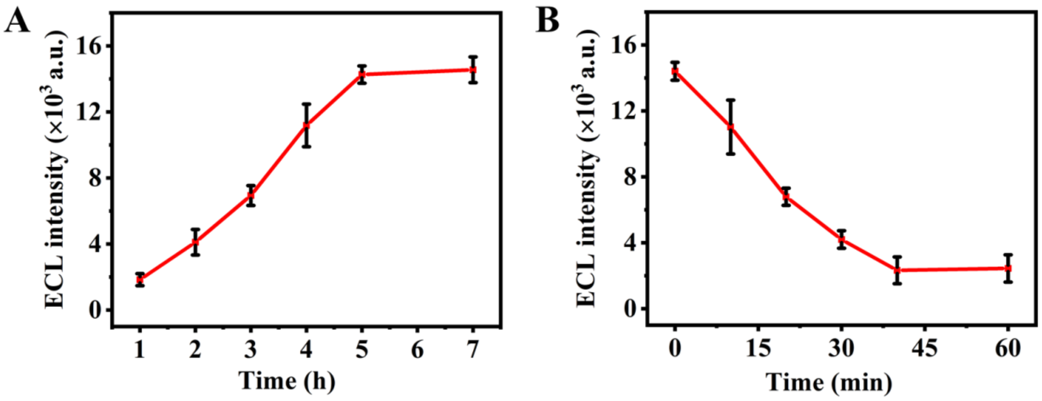

Figure S8 (A) The effect of the self-assembly time of Ir-peptide on the ECL intensity of the ECL biosensor. (B) The effect of the cleavage time of 150 ng·mL^{-1} MMP-3 on the ECL intensity of the ECL biosensor. ECL measurement conditions, 0.10 M PBS (pH 7.40) containing 50 mM TPA. Scan rate, 0.10 V·s^{-1}, PMT, −900 V. Error bars represent the standard deviation of three independent experiments.

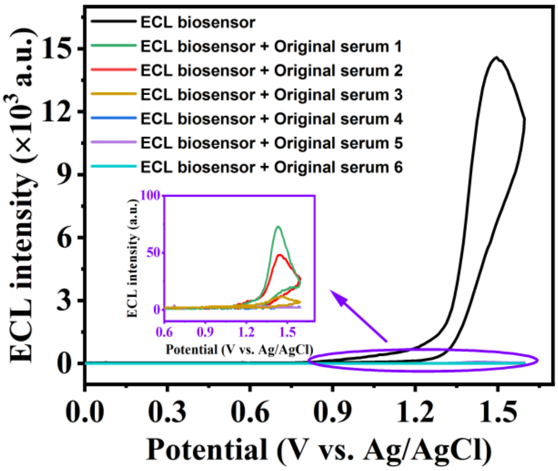

Figure S9. ECL intensity vs. potential profiles of the ECL biosensor in the absence and presence of original serum samples. ECL measurement conditions, 0.10 M PBS (pH 7.40) containing 50 mM TPA. Scan rate, 0.10 V·s^{-1}, PMT, −900 V.

Table S1 Parameter values obtained from the fit of the impedance spectra represented with the equivalent circuit.

Electrode	*R*et (Ω)
Bare GCE	37.93
AuNPs/Nafion/GCE	10,330
Ir-peptide/AuNPs/Nafion/GCE	3106
MCH/zwitterionic peptide@Ir-peptide/AuNPs/Nafion/GCE	6532

Table S2 MMP-3 levels in the serum of patients with rheumatoid arthritis [29].

Disease classification	Concentration of MMP-3 in serum (ng·mL^{-1})
Healthy men	64.5±29.4
Healthy women	29.0±12.7
Early rheumatoid arthritis	246.4±267.7
Late rheumatoid arthritis	224.6±237.3

Table S3 Analytical results of MMP-3 in 20-fold diluted serum of healthy people, measured using ELISA and the developed ECL biosensor.

Samples	ELISA (ng mL^{-1})	Content (ng mL^{-1})	Added (ng mL^{-1})	Found (ng mL^{-1})	Recovery
Healthy People 1	4.1±0.2	/	50	52.8±2.5	105.6%±5.0%
Healthy People 2	3.7±0.3	/	50	46.3±1.4	92.6%±2.8%
Healthy People 3	3.8±0.4	/	50	47.4±3.6	94.8%±7.2%

Table S4 Comparison of different reported methods for the determination of MMP-3.

Method	Linear range	Detection limit	Time for the fabrication of biosensor	Time for the detection of MMP-3	Sample	Ref
Electrochemical immunoassay	0.4-40 ng mL^{-1}	0.4 ng mL^{-1}	22 h	>3.5 h	Serum of healthy people and patients with adrenal cortex carcinoma	[31]
Fluorescence immunoassay	0.73-500 ng mL^{-1}	/	2 h	>85 min	Serum of patients with colorectal cancer	[8]
Fluorescence peptide-based assay	11.4-153.9 ng mL^{-1}	/	>30 h	>2 h	Serum of healthy people, RA and OA patients	[11]
Fluorescence peptide-based assay	108.3-1710 ng mL^{-1}	/	/	>70 min	Collagen-induced arthritis mice serum	[12]
ECL peptide-based assay	10 -150 ng mL^{-1}	8 ng mL^{-1}	5.5 h	<1 h	Diluted serum of healthy people and RA patients	This work
